# Development of a genome-scale metabolic model for the lager hybrid yeast *S. pastorianus* to understand the evolution of metabolic pathways in industrial settings

**DOI:** 10.1128/msystems.00429-24

**Published:** 2024-05-31

**Authors:** Soukaina Timouma, Laura Natalia Balarezo-Cisneros, Jean-Marc Schwartz, Daniela Delneri

**Affiliations:** 1 Manchester Institute of Biotechnology, Faculty of Biology Medicine and Health, University of Manchester, Manchester, United Kingdom; 2 Division of Evolution and Genomic Sciences, School of Biological Sciences, Faculty of Biology Medicine and Health, University of Manchester, Manchester, United Kingdom; LifeMine Therapeutics, Cambridge, Massachusetts, USA

**Keywords:** genome-scale metabolic model, yeast hybrid

## Abstract

*In silico* tools such as genome-scale metabolic models have shown to be powerful for metabolic engineering of microorganisms. *Saccharomyces pastorianus* is a complex aneuploid hybrid between the mesophilic *Saccharomyces cerevisiae* and the cold-tolerant *Saccharomyces eubayanus*. This species is of biotechnological importance because it is the primary yeast used in lager beer fermentation and is also a key model for studying the evolution of hybrid genomes, including expression pattern of ortholog genes, composition of protein complexes, and phenotypic plasticity. Here, we created the iSP_1513 GSMM for *S. pastorianus* CBS1513 to allow top-down computational approaches to predict the evolution of metabolic pathways and to aid strain optimization in production processes. The iSP_1513 comprises 4,062 reactions, 1,808 alleles, and 2,747 metabolites, and takes into account the functional redundancy in the gene-protein-reaction rule caused by the presence of orthologous genes. Moreover, a universal algorithm to constrain GSMM reactions using transcriptome data was developed as a python library and enabled the integration of temperature as parameter. Essentiality data sets, growth data on various carbohydrates and volatile metabolites secretion were used to validate the model and showed the potential of media engineering to improve specific flavor compounds. The iSP_1513 also highlighted the different contributions of the parental sub-genomes to the oxidative and non-oxidative parts of the pentose phosphate pathway. Overall, the iSP_1513 GSMM represent an important step toward understanding the metabolic capabilities, evolutionary trajectories, and adaptation potential of *S. pastorianus* in different industrial settings.

**IMPORTANCE:**

Genome-scale metabolic models (GSMM) have been successfully applied to predict cellular behavior and design cell factories in several model organisms, but no models to date are currently available for hybrid species due to their more complex genetics and general lack of molecular data. In this study, we generated a bespoke GSMM, iSP_1513, for this industrial aneuploid hybrid *Saccharomyces pastorianus*, which takes into account the aneuploidy and functional redundancy from orthologous parental alleles. This model will (i) help understand the metabolic capabilities and adaptive potential of *S. pastorianus* (domestication processes), (ii) aid top-down predictions for strain development (industrial biotechnology), and (iii) allow predictions of evolutionary trajectories of metabolic pathways in aneuploid hybrids (evolutionary genetics).

## INTRODUCTION

Over the last decade, many genome-scale metabolic models (GSMM) of *Saccharomyces cerevisiae* have been constructed (1) and have become increasingly popular as they provide a comprehensive view of the metabolic network and enable the prediction of cellular behavior under different conditions. It successfully helped elucidate new biological processes and design cell factories producing compounds of interest (2). *S. cerevisiae*-related species and hybrids also benefit from a high biotechnological interest. In fact, natural hybrids between *Saccharomyces* species are found in many industrial situations, particularly in beer brewing and in wine making, where hybrid strains evolve due to adaptation to selective environmental conditions (3–5).

*Saccharomyces pastorianus* is an allopolyploid sterile hybrid of the mesophilic *S. cerevisiae* and the cold-tolerant *Saccharomyces eubayanus*. This hybrid species has evolved naturally in industrial settings thanks to its ability to ferment at low temperature (bottom-fermenting lager yeast) under stressful conditions (6). *S. pastorianus* is very efficient to consume and ferment the wide range of sugars found in wort and must, in addition to producing an aroma profile interesting for brewers (7, 8). Specifically, *S. pastorianus* is known for its ability to carry out alcoholic fermentation, converting sugar into ethanol and carbon dioxide, and to produce a range of esters, which are responsible for fruity and floral flavors in beer (9). Besides their industrial importance, *S. pastorianus* can be a key model for studying the evolution of hybrid genomes as this species offers a unique perspective on the genomic alterations that may occur after a recent allopolyploidization event. Comparative genomics analysis highlighted the presence of a complex structural variation among *S. pastorianus* strains, including hybrid genes and evidence of breakpoint re-usage (10, 11). Genomics studies on four *S*. *pastorianus* strains, CBS 1513, CBS 1503, CBS 1538, and WS 34/74, revealed that the low amount of genetic redundancy comes primarily by the loss of the *S. cerevisiae*-like genes (12), and during fermentation, the expression of orthologs is positively correlated with gene copy number (13). It has also been shown that the functional redundancy, generated by the presence of orthologous parental genes, is discouraged, a scenario that fits well with the gene balance hypothesis (14).

Widening the computational modeling methods established for *S. cerevisiae* reference strain to other hybrids is key to study the evolution of hybrid genomes as well as aid media and strain engineering toward the optimization/minimization of desirable/undesirable flavor compounds (15). Evolutionary biology studies are already exploiting genome-scale metabolic models to predict adaptive trajectories in *Escherichia coli*, which could help understand mechanisms behind speciation and adaptation (16, 17). Predictive quantitative approaches have not been used so far with *S. pastorianus* strains due to a lack of molecular data and to the multiplicity of environmental and physiological factors that influence these yeast hybrids during the fermentation process. Indeed, although the sequence of *S. cerevisiae* genome has been available for almost 30 years, only more recently the hybrid genomes of some *S. pastorianus* strains have been sequenced and annotated (10, 12, 18–22). It has been shown that *S. pastorianus* and *S. cerevisiae* exhibit significant differences in gene expression and metabolic levels, and that the Yeast8 model could not always accurately calculate the metabolic flux of *S. pastorianus* (23), hence the need to develop a model specific for *S. pastorianus*.

Here, we present iSP_1513, the first GSMM of the industrial Group I strain *S. pastorianus carlsbergensis* (CBS 1513), which was originally isolated by the Carlsberg Laboratory in Denmark in the late 1800s and is commonly referred to as the “Carlsberg yeast.” This strain is known for its ability to produce clean, crisp, and refreshing lagers with a smooth mouthfeel, and is used by many breweries around the world to produce a wide range of lager styles (24). The iSP_1513 model offers a platform to determine and validate optimal growth conditions for specific traits of industrial relevance, such as maltose utilization, leucine amino acid catabolism (i*.*e*.*, involved in the production of aromatic flavor compound), or ethanol tolerance. Flux balance analysis (FBA) and flux variability analysis (FVA) have been used to predict growth and metabolite production in different environments. The effect of high and low temperatures on metabolite production has been investigated, and a python library has been developed to map transcriptome data. A preference of cellular function between parental alleles has been found, with the oxidative and non-oxidative parts of the pentose phosphate pathway primarily carried out by the *S. eubayanus*-like and *S. cerevisiae*-like genes, respectively. We found that some reactions in this pathway show a shift of parental allele usage according to the temperature. Overall, the iSP_1513 GSMM and the tools developed for this hybrid will aid the prediction of fluxes in metabolic pathways in different environments, ultimately important for strain development, media engineering, and understanding adaptation processes.

## RESULTS AND DISCUSSION

### Genome-scale model draft reconstruction

The Yeast Consensus Model (Yeast8) provides a highly curated and comprehensive representation of yeast metabolism (23, 25) and has been used as a template to reconstruct the draft genome-scale metabolic model of *S. pastorianus*. The reactions in Yeast8 that are present/absent in *S. pastorianus* were identified based on the presence/absence of the genes that support these reactions. Among the 9,728 open reading frames (ORFs) of *S. pastorianus*, 3,742 and 5,219 are *S. cerevisiae*-like genes and *S. eubayanus*-like genes, respectively, while 1,708 pairs of orthologous parental genes are functionally redundant (12).

Firstly, the *S. cerevisiae*-like genes of *S. pastorianus* have been mapped to the genes of Yeast8. Out of the 3,742 *S*. *cerevisiae*-like genes of *S. pastorianus*, 748 were identified as present in Yeast8. Secondly, to map the *S. eubayanus*-like genes of *S. pastorianus* to the Yeast8 genes, one-to-one orthologs between the *S. eubayanus*-like genes and *S. cerevisiae* S288C genes have been searched using the HybridMine software (12). As a result, out of the 5,219 *S*. *eubayanus*-like genes, 2,536 were found to have a one-to-one ortholog in *S. cerevisiae* S288C with more than 70% identity (2,015 shared more than 80%). After this analysis, 622 *S*. *eubayanus*-like genes had a 1:1 ortholog present in the Yeast8 model. In total, 946 out of the 1,160 genes of Yeast8 were found to be also present in *S. pastorianus*, of which 424 have both parental genes, 324 only have an *S. cerevisiae*-like gene associated and 198 only have the *S. eubayanus*-like gene associated, whereas 214 genes present in Yeast8 did not have any 1:1 orthologs in *S. pastorianus*.

The absence of this pool of genes could either be biologically genuine (i*.*e*.*, specific gene loss in *S. pastorianus*) or a technical artefact due an incomplete functional annotation of *S. pastorianus* genes. In fact, the functional annotation was initially performed using the gene sequences of *S. pastorianus* against the gene sequences of the parents *S. cerevisiae* and *S. eubayanus* (12). To strengthen the annotation, the protein sequences were used to search for one-to-one orthologs between the *S. pastorianus* proteins and the two parental proteomes. Following this analysis, we were able to map a further 158 genes from the pool of the 214 Yeast8 genes initially not identified in *S. pastorianus*. As expected, all the *S. cerevisiae*-like and *S. eubayanus*-like genes were also predicted as *S. cerevisiae*-like and *S. eubayanus*-like proteins, respectively. Moreover, we identified inconsistencies between the gene and protein annotation. For example, 29 and 9 *S*. *cerevisiae*-like and *S. eubayanus*-like genes, respectively, had a different annotation associated to their proteins, because HybridMine attributed different isoforms at gene and protein level (Table S1). For instance, the *S. pastorianus* ORF SPGP0D00350 was predicted to be the *S. cerevisiae*-like YPR043W (RPL12A) at the gene level, while at the protein level, it was predicted to be the *S. cerevisiae*-like YJR094W-A (RPL12B). Here, these two paralogs share 100% identity at protein level, hence cannot be discriminated by the software.

**Fig 1:**
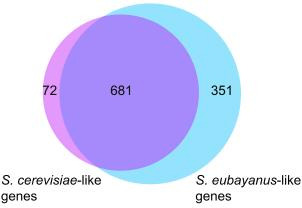
Venn diagrams showing the proportion of *S. cerevisiae*-like (purple) and *S. eubayanus*-like (light blue) genes found in the Yeast8 genome-scale model. The intersection represents the proportion of genes that have both parental copies in *S. pastorianus*.

In summary, out of the 1,160 genes of Yeast8, 1,104 were found to be also present in *S. pastorianus*, of which 681 have both parental genes, 72 only have an *S. cerevisiae*-like gene associated and 351 only have the *S. eubayanus*-like gene associated (Fig. 1). In total, 56 genes were removed as they were not present in *S. pastorianus* genomic data (Table S2), as well as 12 reactions that are exclusively supported by these genes. Additionally, 38 *S*. *pastorianus* genes were removed from the model as they were not associated to any reaction (Table S2). In total, 94 genes were removed from Yeast8.

Gene enrichment was carried out on the 56 *S*. *cerevisiae*-specific genes that were removed from Yeast8. Among them, 10 are involved in transport of sugar (glucose, D-galactose), amino-acid (L-lysine, L-aspartate), ion (thiamine, P-type cation [Cadmium], sodium), and water (Fig. S1). In fact, as membrane proteins are under strong adaptive selection (26), it is not surprising that this class of protein is represented among the proteins specific to *S. cerevisiae* and absent in *S. pastorianus*. Moreover, it has been shown that the gene *SUL1*, encoding a sulfate permease 1, is not functional in *S. pastorianus* (27). This enzyme is indeed present in the list of genes removed (Table S2). Finally, when at least one subunit of a protein complex was not identified as present in *S. pastorianus* genome, the whole protein complex was removed. The list of removed genes includes, indeed, among others, all the genes involved in protein complex supporting mitochondrial reactions, because the mitochondria genome was not represented in the genomic data (i*.*e*.*, please note that the majority of these mitochondrial genes were re-integrated in the model in the follow-up manual curation, see “Genome-scale model manual curation” below). This initial model, named iSP_1513_v0_, represented the first step toward drafting the *S. pastorianus* genome-scale metabolic model.

### Genome-scale model manual curation

Manual curation of a GSMM involves the addition, removal, and modification of reactions and associated genes and metabolites to improve the accuracy and predictive capabilities of the model. Among the reactions that were removed, seven were re-integrated as *S. pastorianus* possesses the enzyme isoforms supporting those reactions (Table S3). In fact, *S. pastorianus* gene SPGP0AH00790 has been predicted at the gene level as *S. eubayanus*-like *HXT5* gene, while the protein was predicted to be Hxt10p. One reaction in Yeast8 involves uniquely Hxt10p, while five reactions involve both isoforms. These six reactions have been re-integrated to iSP_1513 as SPGP0AH00790 can support them. *S. pastorianus* gene SPGP0EQ00100 has been predicted at the gene level as a *S. cerevisiae*-like *FDH2* (truncated protein), while the protein was predicted to be Fdh1p. Only one reaction in Yeast8 involves Fdh1p. Again, as SPGP0EQ00100 can support this reaction, this was re-integrated.

Additionally, among the initial 56 genes removed from Yeast8, the mitochondrial genes *ATP8*, *ATP6*, *COB*, *OLI1*, *COX1*, *COX2*, and *COX3* were present (Table S2), because only the nuclear genomic data of *S. pastorianus* were available. Given that the presence of functional mitochondrial DNA in *S. pastorianus* cells has been confirmed (28), the Yeast8 mitochondrial reactions, initially removed, were investigated to understand whether they could be supported by existing *S. pastorianus* enzymes. As a result, four reactions were re-introduced: (i) as *S. pastorianus* CBS1513 genome contains the *S. eubayanus*-like gene *APA2* (ID: SPGP0Y01780) that supports the ATP adenylyltransferase reaction (ID: r_0222), the reaction and gene were both added to iSP_1513; (ii) the mitochondrial ATP synthase “r_0226” reaction was reintroduced as *ATP6*, *ATP8*, and *OLI1* genes are encoded by the mitochondrion and all the other subunits of this protein complex are present (i*.*e*.*, a total of 18 genes were added to the gene-protein reaction [GPR] of which five have both parental genes); (iii) the Yeast8 mitochondrial ferrocytochrome-c: oxygen oxidoreductase “r_0438” reaction was reintroduced as it is supported by the mitochondrially encoded genes *COX1*, *COX2*, and *COX3* and the other subunits of this protein complex are present (i*.*e*.*, a total of 17 genes were added of which five have both parental genes); and (iv) the Yeast8 mitochondrial ubiquinol: ferricytochrome c reductase “r_0439” reaction was reintroduced as the *COB* gene is encoded by the mitochondrion and the other subunits of this protein complex are present (i*.*e*.*, 11 genes were added of which five have both parental genes).

Next, the reactions specific to *S. pastorianus*, not present in Yeast8, were investigated by mining published data and by employing KBase, a software that offers methods to construct draft genome-scale models. The investigation of published data gave limited insights but clarified a few cases. For example, unlike *S. cerevisiae*, *S. pastorianus* possesses the ability to use melibiose as carbon source, an ability inherited from the *S. eubayanus* parental sub-genome. The alpha-galactosidase (also known as melibiase) required to break the melibiose into a glucose and a galactose is encoded by the gene *MEL1*, and is not present in Yeast8. The new reaction (ID: r_4711), confirmed as balanced by eQuilibrator (29), as well as a new gene (ID: SPGP0R03440; Name: MEL1_Seub), were added to iSP_1513.

Next, we built an independent *S. pastorianus* GSMM draft using KBase. The resulting model was manually updated to take into account the functional redundancy of *S. pastorianus*. This updated model contains 1,206 reactions, 1,228 metabolites, and 1,207 genes. The *S. pastorianus* genes and reactions that are present in the KBase draft but absent from the draft iSP_1513 _v0_ were identified. Because of the heterogeneity of reaction names between the iSP_1513 _v0_ and the KBase models, regular expressions were used to catch keywords within reaction names. As a result, 1,046 and 45 KBase reactions and genes, respectively, were found to be absent from iSP_1513 _v0_, and therefore could be potentially added (Table S4). Out of the 45 genes, 23 are *S. cerevisiae*-like alleles, 17 are *S. eubayanus*-like alleles, and 5 *S*. *pastorianus* specific. The reactions supported by these genes were mapped to the iSP_1513 _v0_ GSMM to identify genes/reactions specific to *S. pastorianus*. In total, 5, 26, and 3 reactions, genes, and metabolites were added, respectively. The reactions/genes are detailed in File S1.

### GPR rules update for iSP_1513

Because *S. pastorianus* carries an aneuploid and hybrid genome, genes can have *S. cerevisiae*-like alleles and *S. eubayanus*-like alleles, which results in functional redundancy. Therefore, the GPR rules of Yeast8 are not applicable to *S. pastorianus* as this functional redundancy needs to be considered to enable gene deletion simulations or integration of gene expression data. Metabolic reactions can be non-enzymatic, when catalyzed by small molecules, or enzymatic, when catalyzed by specific proteins (30). From a structural point of view, enzymes can be either monomeric, meaning they are composed of a single gene product, or oligomeric, meaning they are composed of multiple gene products. In the context of predicting the effects of gene deletion on metabolic fluxes, it is important to consider functional redundancy in the genome-scale models, including type and composition of catalysts. This is particularly relevant in hybrid species where both parental genes are competing. GPRs are Boolean logic relationships between gene products, such as enzyme isoforms or subunits, involved in catalyzing a particular reaction. The presence of functionally redundant parental alleles may impact on the nature of protein complexes established in the hybrid, where both parental alleles are competing. The protein complexes in *S. pastorianus* can be either exclusively uni-specific with subunits coming only from one parent, or exclusively chimeric, with a mixture of subunits from both parents, or partially or fully redundant when a series of protein complexes with different orthologous members can be established because both alleles are present for some or all subunits, respectively (14). This way, biologically meaningful phenotypic predictions can be derived as a function of gene expression profiles encoding for subunits or isoforms of the involved enzymes. Regardless of the used approach to integrate omics data, the reliability of the formulated hypotheses strongly depends on the quality of GPR rules included into the models, which describe how gene products concur to catalyze the associated reactions.

The *S. cerevisiae* gene IDs in the GPR rules of the iSP_1513 _v0_ GSMM were replaced by the *S. pastorianus* parental alleles ID. In instances where a gene possessed both parental variants, a principle was established to acknowledge that either of the parental proteins could facilitate the reaction, given their functional redundancy. To accomplish this, an "OR" operator was employed to link genes that encoded different protein isoforms of the identical enzyme or subunit. The gene names were replaced with the common gene name combined with “_scer,” “_seub,” or “_spast” to inform whether they are *S. cerevisiae*-like, *S. eubayanus*-like, or *S. pastorianus*-specific genes, respectively. This curated model, named iSP_1513, represents the *S. pastorianus* genome-scale metabolic model.

The majority of the reactions are associated with genes via GPR associations. In total, there are 762 reactions that are supported by only one gene, 1,694 reactions that are supported by functionally redundant genes (Boolean "OR"), 102 reactions that are supported by a protein complex (Boolean "AND"), and 105 reactions that are supported by a combination of genes and protein complexes (Boolean "AND" and "OR"). However, there are also reactions that are not associated with any genes. In fact, there are 1,399 reactions that are not associated with any genes of which 265 are exchange reactions. This information can be useful for understanding the genetic basis of metabolic pathways and for designing strategies for manipulating metabolic networks.

### Quality control analysis

iSP_1513 genome-scale model was evaluated using MEMOTE software (31), which uses a standardized and community-maintained set of metabolic model tests. The overall score of the iSP_1513 model is 68%, against 69% for Yeast8.6.2 model. The model was determined to have a 50% consistency, 68% metabolite annotations, 65% reaction annotations, 52% gene annotations, and 88% Systems Biology Ontology (SBO) term annotations.

The full MEMOTE report for iSP_1513 can be accessed at https://github.com/Sookie-S/Spastorianus_GSMM_iSP_1513/.

### Simulations of the metabolism of *S. pastorianus* using iSP_1513 and Yeast8

In *S. cerevisiae*, a large-scale study found that approximately 20% of the genes are essential. These genes are involved in a variety of cellular processes, including DNA replication, protein synthesis, and cell division and metabolism (32). In comparison, less is known about essential genes in *S. pastorianus*, as this species has not been subjected to a large functional analysis. For the genes involved in the metabolism, we carried out single knockout simulations using iSP_1513 and Yeast8, and predicted 42 essential and 126 essential genes in synthetic defined (SD) medium with 2% glucose, respectively. The lower number of essential genes predicted by iSP_1513 reflects the redundancy present in *S. pastorianus*, which is not captured by Yeast8. To show the importance to take into account genetic redundancy after hybridization, we experimentally validated two genes (four orthologs), which are essential in *S. cerevisiae* but non-essential in *S. pastorianus. S. cerevisiae*-like and *S. eubayanus*-like genes of known *S. cerevisiae* essential genes, namely *FOL1* (i*.*e*.*, involved in folic acid synthesis) and *BPL1* (i*.*e*.*, encoding for a biotin protein ligase), have been deleted separately in *S. pastorianus* using long flanking PCR-mediated gene replacement. The single mutant strains were able to survive in rich medium, such as yeast extract–peptone–dextrose(YPD), indicating that the presence of one functional gene coming from either the *S. cerevisiae* or the *S. eubayanus* parent is enough to compensate for the loss of the other (Fig. S2). Analysis of single reaction knockout indicated that both models have the same 205 essential reactions (Table 1 and Table S5). Overall, we found variation in the prediction of essential genes, but not reactions, between iSP_1513 and Yeast8.

**TABLE 1:** Predicted number of essential genes and reactions in SD medium + 2% glucose in *S. pastorianus* and *S. cerevisiae*.

Model	Essentiality	SD + 2% glucose
iSP_1513	Essential genes	42
	Essential reactions	205
Yeast8	Essential genes	126
	Essential reactions	205

**Fig 2:**
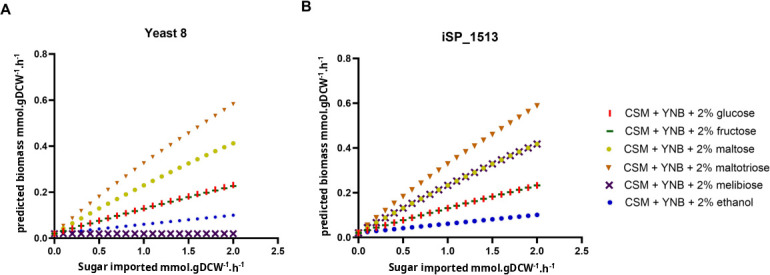
Predicted biomass (millimoles per gram of dry cell weight per hour) according to the sugar uptake. A sugar import of 20 represents the total consumption of the sugar present in the SD medium. Panel A, predictions using Yeast8; panel B, predictions using iSP_1513.

We then validated our model for growth on different sugars. We predicted the growth of *S. pastorianus* using both iSP_1513 and Yeast8 in SD complete medium with different carbon sources, by performing FBA with the objective function set to growth optimization. The iSP_1513 model showed biomass production in all the sugars. In glucose, maltose, maltotriose, and ethanol, iSP_1513 and Yeast8 showed similar biomass produced. In medium containing melibiose as sole carbon source, no biomass was detected using Yeast8, while our model was able to predict growth (Table 2; Fig. 2). In fact, *S. pastorianus* has been experimentally shown to be able to utilize melibiose as a carbon source for growth and fermentation, while *S. cerevisiae* is not able to support growth on this sugar alone (33), although some *S. cerevisiae* strains have been shown to metabolize melibiose to some extent (34). Hence, it is important to note that the growth and biomass yields of *S. pastorianus* on different sugar sources can be influenced by many factors, including the specific strain of yeast used, the composition of the culture media, and the environmental conditions of the fermentation process.

**TABLE 2:** Predicted biomass of *S. pastorianus* using iSP_1513 and Yeast8 in different media

Medium	Biomass (mmol · g DCW^−1^ · h^−1^) using:	
	iSP_1513	Yeast8
SD	0.0204	0.0197
SD + 2% glucose	0.2325	0.2315
SD + 2% maltose	0.4172	0.4125
SD + 2% maltotriose	0.5884	0.5827
SD + 2% melibiose	0.4172	0.0197
SD + 2% ethanol	0.1006	0.0999

The iSP_1313 GSMM constrained using transcriptome data obtained in SD medium at 13°C, 22°C, and 30°C predicted 0.20 h^−1^, 0.23 h^−1^, and 0.22 h^−1^ growth rates, respectively. The measured growth rates in complete SD medium at 13°C, 22°C, and 30°C are 0.12 h^−1^, 0.22 h^−1^, and 0.20 h^−1^, respectively (14). Both prediction and experimental data show the highest growth rate around *S. pastorianus* optimal temperature of growth (22°C), and the lowest growth rate at 13°C. However, at 13°C, iSP_1513 overestimated the growth rate, suggesting that mapping the transcriptome data is not enough to capture effects of cold on growth rate. This prediction could be improved once other ’omics data, such as proteomics, are added (35).

### iSP_1513 metabolite secretion simulations in SD medium with various leucine content

The secretion of 2-phenyl ester (roses, honey, apple, sweet flavors), isoamyl acetate (banana and pear flavors), ethyl decanoate (floral and fruity flavors), and ethyl octanoate (apple, tropical fruit, sweet flavors) were investigated in SD medium with different concentrations of leucine.

Regardless of the presence or absence of leucine, the iSP_1513 model predictions indicate that *S. pastorianus* can produce 2-phenyl ester, ethyl octanoate, ethyl decanoate, and isoamyl acetate in a minimal media (Table 3). *S. pastorianus* possesses inherent metabolic capabilities that enable enhanced synthesis of these compounds. Two genes, *ATF1* and *ATF2*, encoding for alcohol acetyltransferases (AATase, EC 2.3.1.84), are responsible for synthesizing acetate esters, including isoamyl acetate, through the reaction between acetyl coenzyme A and their respective alcohols (36). These esters play a significant role in shaping the flavor profiles of beer and various other alcoholic beverages. *S. pastorianus* CBS 1513 possesses a *S. eubayanus*-like *ATF1* gene (SPGP0DZ02280) and both *S. cerevisiae*-like and *S. eubayanus*-like *ATF2* genes (SPGP0F01100 and SPGP0Q01180, respectively).

**TABLE 3:** Predictions of metabolite secretion in SD medium with various leucine content using flux variability analysis (objective set to growth, fraction of optimum 90%)

Media	iSP_1513 predicted metabolite production (mmol · g DCW^−1^ · h^−1^)			
	2-Phenylethyl ester	Isoamyl acetate	Ethyl octanoate	Ethyl decanoate
SD media	0.1227	0.2113	0.0661	0.0595
SD media without leucine	0.1248	0.1558	0.0672	0.0605
SD media with extra leucine	0.1231	0.2828	0.0663	0.0598

**Fig 3:**
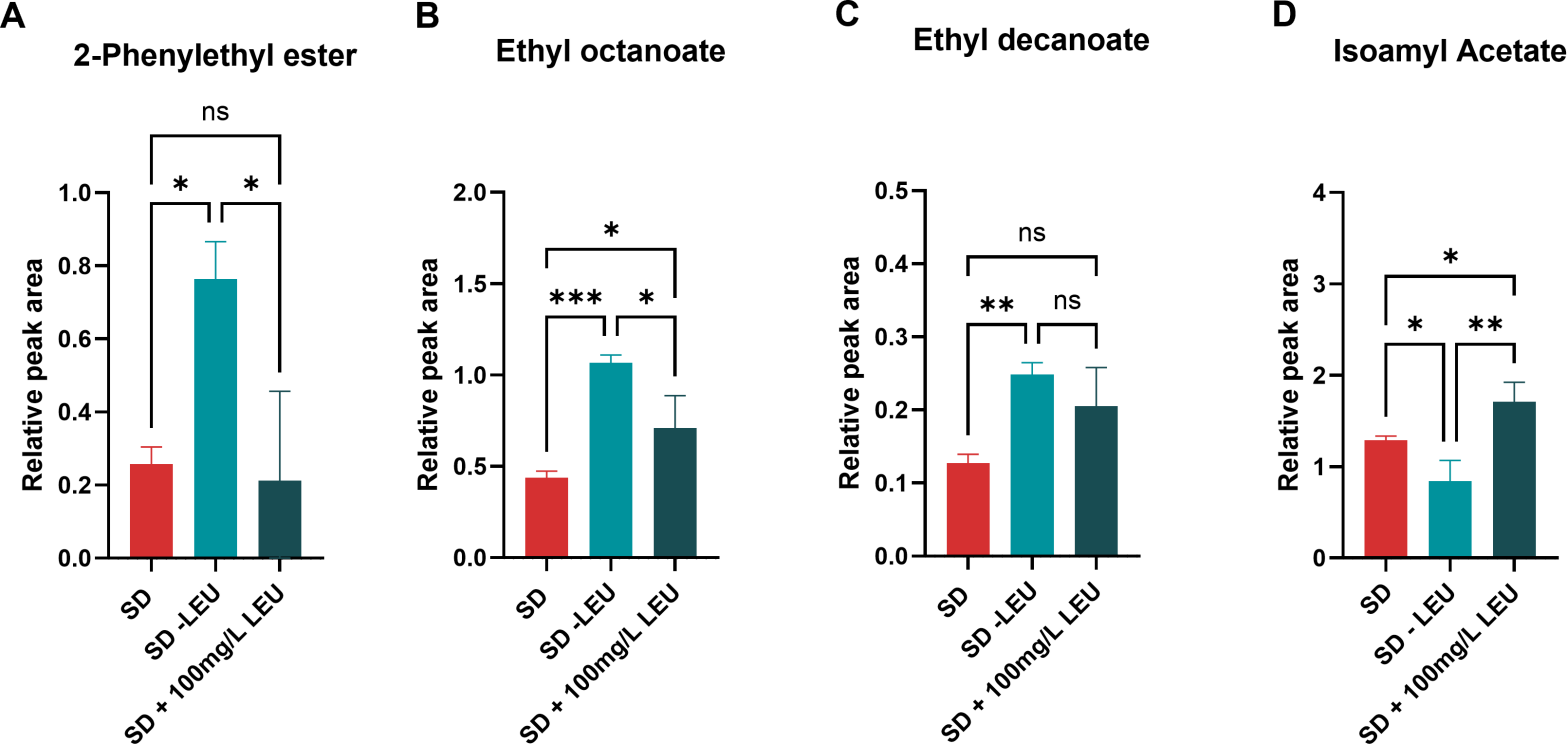
*S. pastorianus* CBS 1513 metabolite production in SD medium, SD without leucine, and SD with an additional 100 mg/L leucine measured by GC-MS for the metabolites: panel A, 2-phenyl ester; panel B, ethyl octanoate; panel C, ethyl decanoate; panel D, isoamyl acetate. Symbols: *, *P*-value ≤ 0.05, **, *P*-value ≤ 0.01; ***, *P*-value ≤ 0.001; ns, nonsignificant.

The absence of leucine in the medium is predicted by the model to have an enhancing effect on the production of 2-phenyl ester, ethyl octanoate, and ethyl decanoate in *S. pastorianus*. Furthermore, higher concentrations of leucine in the medium are predicted to correspond to an increased production of isoamyl acetate. These predictions have been validated by quantifying these compounds, via gas chromatography-mass spectrometry (GC-MS), in *S. pastorianus* cultures grown on SD, SD with an additional 100 mg/L leucine, and SD without leucine. When comparing the volatiles produced in the different media, we found a significantly higher amount of 2-phenyl ester, ethyl octanoate, and ethyl decanoate produced when *S. pastorianus* was grown on SD-Leu (Fig. 3A through C), and a significantly higher amount of isoamyl acetate when the medium was supplemented with leucine (Fig. 3D). The increase of isoamyl acetate can be attributed to leucine’s role as a precursor in the biosynthesis of isoamyl acetate via the Ehrlich pathway. The availability of leucine in the medium likely promotes the enzymatic conversion of leucine to isoamyl acetate, resulting in higher levels of isoamyl acetate production. Overall, the amount of leucine in the extracellular medium may lead to a redirection of metabolic pathways, promoting or suppressing the synthesis of these volatiles. Such scenario has been correctly predicted by our model and highlights the ability of iSP_1513 to be used in biotechnology for media engineering when growing *S. pastorianus*.

### Integration of transcriptome data to investigate the impact of temperature on metabolite production

The mapping of transcriptome data to constrain genome-scale metabolic model reactions allows the integration of additional parameters for the simulations, such as temperature. The GSMM also provides a scaffold for the identification and visualization of the contribution of each parental allele to metabolic pathways, uncovered by the transcription data. We have transcriptome data of *S. pastorianus* CBS1513 cultured in SD complete medium and SD medium lacking leucine at 13°C, 22°C, and 30°C (14).

By mapping the normalized read counts onto the GSMM from the culture grown in SD medium at 13°C, 22°C, and 30°C, we found that 1,107, 1,139, and 1,221 iSP_1513 reactions have been constrained, respectively; while for the cultures grown in SD medium lacking leucine at 13°C, 22°C, and 30°C, we found that 1,102, 1,175, and 1,170 iSP_1513 reactions have been constrained, respectively.

**Fig 4:**
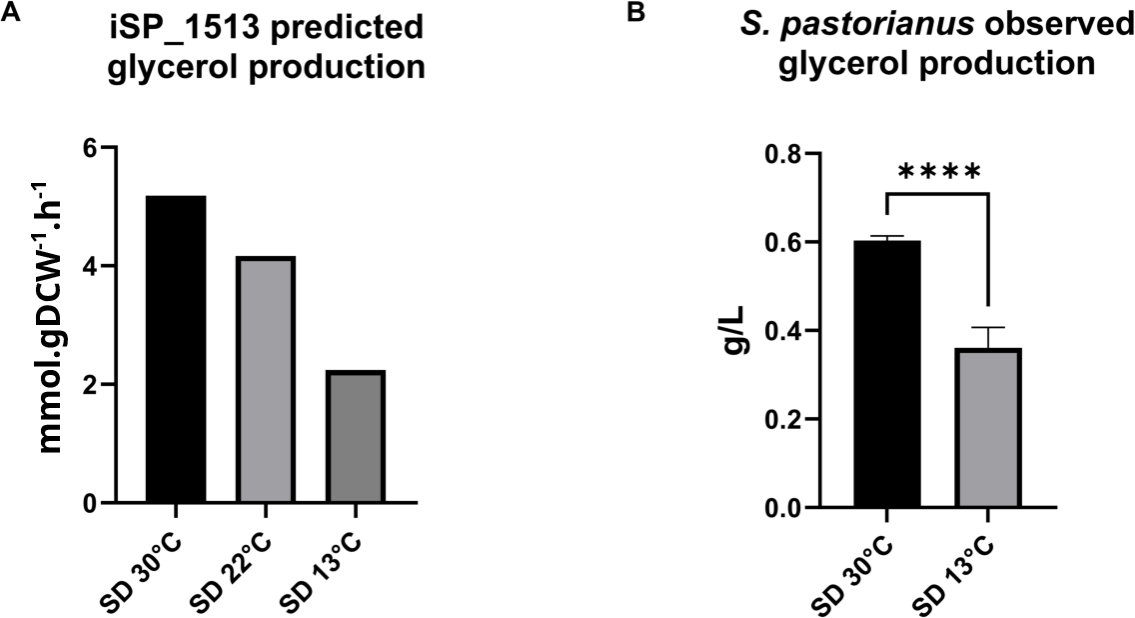
*S. pastorianus* CBS 1513 glycerol production in SD medium as predicted by the iSP_1513 model constrained using the following: panel A, the transcriptome data obtained in SD medium at 13 °C, 22°C, and 30°C; panel B, as measured experimentally with HPLC in SD medium at 13°C and 30°C. Symbols: ****, *P*-value ≤ 0.0001.

To showcase the usefulness of this approach, using the iSP_1513, we predicted the glycerol production at 13°C and 30°C in SD medium, and we experimentally validated the predictions. Glycerol is important for the maintenance of membrane integrity during temperature shifts. At hot temperatures, yeast cells increase glycerol production as a protective mechanism against heat stress, while at cold temperatures, they decrease glycerol production to maintain proper membrane fluidity (37). Overall, the regulation of glycerol production in yeast is dependent on the temperature, which acts as a crucial factor in maintaining membrane functionality under varying environmental conditions. In SD medium, iSP_1513 predicted that glycerol production reaches its peak at 30°C, whereas the lowest levels are detected at 13°C (Fig. 4A). To validate these predictions, small-scale fermentations were performed at 13°C and 30°C in SD medium. As predicted, when the glucose is fully consumed, the production of glycerol was found to be twofold higher at 30°C compared to 13°C (Fig. 4B). The utilization of transcriptome data mapping to constrain the genome-scale metabolic model reactions yielded a precise forecast of glycerol production while affirming the inclusion of temperature as a parameter in the analysis.

**Fig 5:**
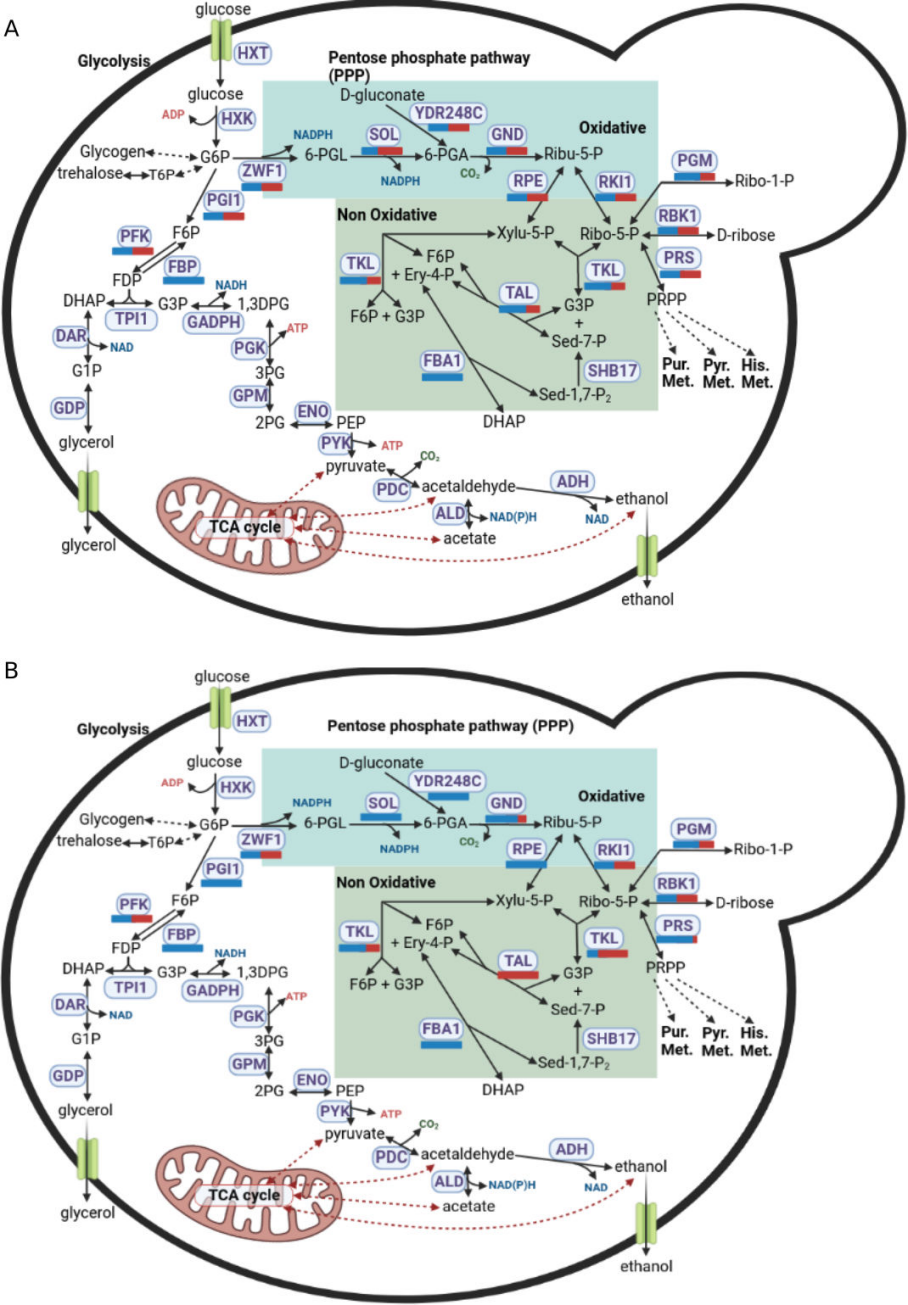
Carbon central metabolism in *S. pastorianus*. Panel A, proportion of *S. eubayanus*-like and S. *cerevisiae*-like genes present in the genome supporting each reaction. Panel B, proportion of *S. eubayanus*-like and S. *cerevisiae*-like genes expressed at 22 °C in SD medium. *S. eubayanus*-like and S. *cerevisiae*-like genes are represented as blue and red bars, respectively.

The GSMM also provides a scaffold for the identification and visualization of the contribution of each parental allele to metabolic pathways, uncovered by the transcription data. The integration of transcriptome data to investigate the parental sub-genome activity is particularly important for hybrid species, where both parental gene copies are present and, according to the environmental conditions, only one parental gene may be expressed (14). In SD medium at 22°C, we found that for the pentose phosphate pathway, although both parental genes can carry the reactions (Fig. 5A), there is a preference of cellular function between parental alleles, with the oxidative and non-oxidative parts of pathway primarily carried out by the *S. eubayanus*-like and *S. cerevisiae*-like genes, respectively (Fig. 5B; Table S6). These results hold true also at 13°C and 30°C (Table S6). Some reactions show that one of the two alleles is predominant in all the temperatures. For example, the phosphogluconate dehydrogenase reaction, supported by *GND1* or *GND2* (both having *S. cerevisiae*-like and *S. eubayanus*-like alleles), is carried out at 80%, 85%, and 90% by the *S. eubayanus*-like alleles at 13°C, 22°C, and 30°C, respectively (Table 4). Other key metabolic reactions show a shift of parental allele usage according to the temperature. For example, the ribokinase reaction, supported by *RBK1*, is equally supported by both parental alleles at 13°C and 22°C, while the *S. cerevisiae*-like alleles are primarily expressed at 30°C (Table 4). The phosphopentomutase reactions, supported by *PGM1* (*S. eubayanus*-like alleles), *PGM2* (both parental alleles), or *PGM15* (both parental alleles), are equally supported by both parental alleles at 13°C, while at 22°C and 30°C, there is a shift of expression toward the *S. eubayanus*-like alleles (Table 4). Finally, the phosphofructokinase reactions, supported by a protein complex composed with the product of *PFK1* (both parental alleles) and *PFK2* (both parental alleles), are mainly supported by the *S. eubayanus*-like alleles at 13°C, while at 30°C, it is equally supported by both parental alleles (Table 4).

**TABLE 4:** Proportion of *S. eubayanus*-like and *S. cerevisiae*-like genes supporting the reactions of the pentose phosphate pathway in SD medium at 13°C, 22°C, and 30°C

Reaction name	GSM reaction GPR	Proportion of genes supporting the reaction in SD media at 13°C		Proportion of genes supporting the reaction in SD media at 22°C		Proportion of genes supporting the reaction in SD media at 30°C	
		*S. cerevisiae*-like genes	*S. eubayanus*-like genes	*S. cerevisiae-*like genes	*S. eubayanus*-like genes	*S. cerevisiae*-like genes	*S. eubayanus*-like genes
Glucose-6-phosphate isomerase	PGI1_Scer or PGI1_Seub	0%	100%	0%	100%	0%	100%
Glucose-6-phosphate dehydrogenase	ZWF1_Scer or ZWF1_Seub	Inconclusive	Inconclusive	Inconclusive	Inconclusive	Inconclusive	Inconclusive
6-Phosphogluconolactonase	SOL1_Scer or SOL1_Seub or SOL2_Scer or SOL3_Scer or SOL3_Seub or SOL4_Scer or SOL4_Seub	0%	100%	0%	100%	0%	100%
Phosphogluconate dehydrogenase	GND1_Scer or GND1_Seub or GND2_Scer or GND2_Seub	20%	80%	15%	85%	10%	90%
Ribulose 5-phosphate 3-epimerase	RPE1_Scer or RPE1_Seub	0%	100%	0%	100%	0%	100%
Transketolase	TKL1_Seub or TKL2_Scer or TKL2_Seub	85%	15%	85%	15%	85%	15%
Transaldolase	TAL1_Seub or NQM1_Scer or NQM1_Seub		100, 0		100, 0		100, 0
Ribose-5-phosphate isomerase	RKI1_Scer or RKI1_Seub	Inconclusive	Inconclusive	Inconclusive	Inconclusive	Inconclusive	Inconclusive
Ribokinase	RBK1_Scer or RBK1_Seub	50%	50%	50%	50%	55%	45%
Phosphoglucomutase	PGM1_Seub or PGM2_Scer or PGM2_Seub	Inconclusive	Inconclusive	Inconclusive	Inconclusive	Inconclusive	Inconclusive
Phosphopentomutase	PGM1_Seub or PGM2_Scer or PGM2_Seub or PRM15_Scer or PRM15_Seub	50%	50%	40%	60%	30%	70%
Phosphoribosylpyrophosphate synthetase	(PRS1_Seub and (PRS2_Scer or PRS2_Seub)) or (PRS1_Seub and (PRS3_Scer or PRS3_Seub)) or (PRS1_Seub and (PRS4_Scer or PRS4_Seub)) or ((PRS2_Scer or PRS2_Seub) and (PRS5_Scer or PRS5_Seub)) or ((PRS4_Scer or PRS4_Seub) and (PRS5_Scer or PRS5_Seub))	5%	95%	5%	95%	5%	95%
ATP:D-gluconate 6-phosphotransferase	YDR248C_Scer or YDR248C_Seub	0%	100%	0%	100%	0%	100%
Fructose-bisphosphate aldolase	FBA1_Seub	Inconclusive	Inconclusive	Inconclusive	Inconclusive	Inconclusive	Inconclusive
Fructose-bisphosphatase	FBP1_Seub	0%	100%	0%	100%	0%	100%
Phosphofructokinase	((PFK1_Scer or PFK1_Seub) and (PFK2_Scer or PFK2_Seub))	40%	60%	45%	55%	50%	50%

### Conclusions

The development of iSP_1513 represents a significant advancement in modeling the metabolism of hybrid yeast strains and enhances industrial approaches to fermentation processes. Despite the challenges posed by the lack of molecular data and the complexity of physiological features of yeast aneuploid hybrids, recent progress in genome sequencing and annotation has made possible the construction of genome-scale metabolic models for specific *S. pastorianus* strains.

iSP_1513, the first GSMM tailored to the industrial strain *S. pastorianus* CBS 1513, addresses the issue of functional redundancy caused by orthologous parental alleles. This unique characteristic enables accurate predictions of gene knockouts and omics data mapping. Notably, contrary to the Yeast8 model, iSP_1513 is able to utilize melibiose as a carbon source. Moreover, iSP_1513 predited that *S. pastorianus* produces a higher amount of 2-phenyl ester, ethyl octanoate, and ethyl decanoate flavor compounds when grown on SD medium lacking leucine, and a significantly higher amount of isoamyl acetate when the medium was supplemented with leucine.

We developed a universal Python3 function as a library, crucial for mapping transcriptome data onto GSMM reactions, hence facilitating growth predictions under various environmental conditions including temperature as a parameter. iSP_1513 correctly predicted that the production of glycerol increases when the external temperature increases (i*.*e*.*, in accordance with the experimental validation). In SD medium, we found that there is a preference of cellular function between parental alleles, with the oxidative and non-oxidative parts of the pentose phosphate pathway primarily carried out by the *S. eubayanus*-like and *S. cerevisiae*-like genes, respectively. For some reactions of this pathway, a shift of parental allele usage according to the temperature was observed.

Overall, iSP_1513 represents a significant step forward in our ability to model the metabolism of hybrid yeast strains and optimize industrial fermentation processes. The iSP_1513 model provides a platform for predicting optimal growth conditions in different environments, thanks to the inclusion of the functional redundancy of parental alleles in enzymatic reactions within the gene protein rules of the model. By employing FBA and FVA, iSP_1513 can predict growth in diverse environments and is capable of anticipating reactions that promote or suppress the production of specific aroma compounds. This feature enables more efficient and tailored fermentation processes, making it a valuable tool in the field of biotechnology and industrial bioprocessing. Ultimately, the iSP_1513 model can be used in combination with evolutionary algorithms to predict adaptation trajectories under different environmental pressures, and hence provide a top-down framework to study genome evolution in hybrids.

## MATERIALS AND METHODS

### Genome sequence and annotation

*S. pastorianus* CBS 1513 strain has been sequenced and assembled (10, 19). Its genome sequence is available from the National Center for Biotechnology Information (NCBI: txid1073566). The Yeast Genome Annotation Pipeline (YGAP) has been used to predict the potential ORFs in its genome (38). HybridMine tool (https://github.com/Sookie-S/HybridMine, v4.0) has been used to identify the parental allele content in this strain (12). Among the 9,728 potential ORFs of *S. pastorianus* CBS 1513, there are 5,218 *S*. *eubayanus*-like alleles and 3,739 *S*. *cerevisiae*-like alleles, of which 1,706 are ortholog alleles (12). To enrich the pool of *S. pastorianus* gene annotated, one-to-one orthologs were searched between *S. pastorianus* predicted proteins and the proteome of the two parental species, using HybridMineP (https://github.com/Sookie-S/HybridMineP). HybridMineP is using the same pipeline as HybridMine except for using BLASTP instead of BLASTN for finding one-to-one orthologs between the species.

### GSMM draft reconstruction

The COBRApy package has been used in a Python 3.6 environment to reconstruct the genome-scale metabolic model. The yeast consensus genome-scale model Yeast8 version 8.6.2 (25) has been used as a template to construct a draft genome-scale model of *S. pastorianus* CBS1513. Yeast8 contains 1,160 genes, 2,744 metabolites, and 4,063 reactions. First, the *S. cerevisiae*-like alleles of *S. pastorianus* were mapped. Next, one-to-one orthologs were searched between the *S. eubayanus*-like alleles of *S. pastorianus* and the *S. cerevisiae* S288C genes using HybridMine software (12). The GPR associations, which are Boolean statements connecting genes to reactions, were updated to take into account the ortholog parental alleles found in *S. pastorianus*. The same biomass reaction of Yeast8 was used in the iSP_1513 model. The Gprofiler tool (39) was used to conduct a gene ontology enrichment analysis on the removed *S. cerevisiae*-specific genes.

Additionally, to identify *S. pastorianus*-specific reactions, a draft GSMM reconstruction was generated through the KBase (40) narrative interface. *S. pastorianus* CBS1513 (NCBI: txid1073566) genome in FASTA format was uploaded into the staging area and, subsequently, imported into the narrative through the “Import FASTA File as Assembly From Staging Area” app. Its genome annotation file (12) in GFF3 format was uploaded into the staging area and, subsequently, imported into the narrative through the “Import GFF3/FASTA File as Genome From Staging Area” app. Draft metabolic reconstructions were generated through the “Build Fungal Model” app and exported in Systems Biology Markup Language (SBML) format through the “Bulk Download Modeling Objects” app. KBase draft GSMM does not take into account the gene redundancy, the redundant orthologous alleles were added to the reaction GPR rule using an in-python script. The *S. pastorianus* genes and reactions that are present in the KBase GSMM draft but absent from iSP_1513 were identified. Genes in the KBase model that are absent in the iSP_1513 draft were searched as they could be potentially added. The reactions supported by these genes were mapped to the iSP_1513 GSMM to identify genes/reactions specific to *S. pastorianus*. With this methodology, the genes/reactions were added to iSP_1513. The eQuilibrator database was used to verify the balance of the added reactions (29). SBO terms were added to the new genes, metabolites, and reactions. MEMOTE (31) was used to perform model quality-control analysis in a standardized manner.

The *S. pastorianus* model has been named iSP_1513 and is encoded in the SBML.

### Model simulations

A metabolic flux corresponds to the amount of a metabolite processed by one or more catalytic steps per unit of time, normalized by cellular abundance. Metabolic fluxes have a unit of millimoles per gram of dry cell weight per hour (mmol · g DCW^−1^ · h^−1^) (41). Simulation using stoichiometric models allows a quantitative understanding of metabolism. FBA calculates the flow of metabolites through a metabolic network; therefore, the growth rate of an organism or the production rate of a metabolite can be predicted, under the assumption that during exponential growth, the metabolic function produces a constant flux of biomass (42). Steady-state mass balance is assumed. FBA is concerned with the following linear program (LP):


(1) $$\begin{array}{ll} max_{v} & cT\cdot v\\ subject\;to & S\cdot v=0\\ v_{l}\leq v\leq v_{u} & \end{array}$$

where *S* is an *m · n* stoichiometric matrix of a metabolic network with *m* metabolites and *n* reactions, and *c* is the vector representing the linear objective function; *v* is the rate of metabolic flux, with *V* ⊆ ℝ^n^; the vectors *v_l_* and *v_u_* represent the flux of the lower and upper bounds, respectively. The upper bounds were set as 1,000 while the lower bounds were set as −1,000 for the reversible reactions, and 0 for the irreversible reactions. The substrate uptake reaction (such as the consumption of glucose, oxygen, or ammonia) is changed to a specific value according to the medium of cell growth. The constraints *S . v* = 0 together with the upper and lower bounds specify the feasible region of the problem.

Although FBA only finds the maximum flux for the model reactions, FVA (43) calculates the minimum and maximum flux for the reactions while maintaining some state of the network, e.g., supporting 90% of maximal possible biomass production rate. FVA allows the exploration of alternative optima of equation 1. Let *w* represent some biological objective such as the biomass production. After solving equation 1 with *c* = *w*, FVA solves two optimization problems for each flux *v_i_* of interest:


(2) $$\begin{array}{rl} & max_{v}/min_{v}\;v_{i}\\ & subject\;to\;S\cdot v=0\\ & wT\cdot v\geq \gamma \cdot Z0\\ & v_{l}\leq v\leq v_{u} \end{array}$$

where *Z*0 = *wT · v*0 is an optimal solution to equation 1, *γ* is a parameter, which controls whether the analysis is done with regard to suboptimal network states (0 ≤ *γ* < 1) or to the optimal state (*γ* = 1). Assuming that all n reactions are of interest, FVA requires the solution of 2n LPs.

FBA and FVA simulations were performed using COBRApy package (v. 0.18.1). The IBM ILOG CPLEX solver has been used to find the optimal and sub-optimal solutions. FBA and FVA were performed on conditions reflecting SD medium + 2% glucose (Table S7). SD medium composition was taken from Harrison et al. (44), and the substrate intake fluxes were updated to model the SD medium purchased from Formedium that was used experimentally.

### Transcriptome data processing

Gene expression data of *S. pastorianus* CBS1513 grown in SD medium + 2% glucose and SD medium without leucine + 2% glucose at 13°C, 22°C, and 30°C were taken from Timouma et al. (14). The raw read counts of the RNAseq data were normalized for sequencing depth and gene length using the transcripts per kilobase million (TPM) method, using the bioinfokit python 3 library. The read counts were divided by the length of each gene in kilobases, which gives reads per kilobase (RPK) normalization. All the RPK values in a library were counted up, and the result was by 1,000,000, which gives the “per million” scaling factor. Finally, the RPK values were divided by the “per million” scaling factor, which gives the TPM. The advantage of this method is that the sum of all TPMs in each library is the same and that the genes within a library can be compared.

### Mapping transcriptome data to constrain GSMM reactions

To integrate temperature as a parameter in addition to the media composition, gene expression data of *S. pastorianus*, normalized with the TPM method, were mapped to the metabolic reactions following the methodology described in Lee et al. (45). This methodology relies on the hypothesis that metabolic fluxes are influenced by the levels of enzymes and/or their corresponding transcripts (46). The Boolean "OR" relationship signals alternative catalysts so the total capacity of the reaction is given by the sum of its components, while the Boolean "AND" relationship signals a complex between several gene products; therefore, the maximum complex concentration is given by the minimum concentration of its components. The "AND" Boolean relationship is above the "OR" relationship within the GPR. Irreversible reactions will have their upper bound constrained with the positive final expression value, while reversible reactions will have both their upper bound and lower bound constrained with the positive and negative final expression values, respectively (45).

We developed a universal python 3 function, “map_transcriptome_data,” which can be broadly used for any genome-scale model. First, “map_transcriptome_data” analyzes the GPR associations in a model of metabolic reactions. The function categorizes the GPR associations into several groups based on their Boolean relationships, such as "OR", "AND", and a mixture of both. The reactions for which the GPR that is supported by only one gene will be constrained using the expression data of the gene. The GPRs that contain only the "OR" Boolean relationship will be constrained by the sum of the expression data of the genes. The GPRs that contain only the "AND" Boolean relationship will be constrained by the minimal expression data of the genes. The GPR that contains a mixture of "OR" and "AND" Boolean relationships is the most complex case; therefore, a recursive function was implemented within the “map_transcriptome_data” function to tackle these cases by order of priority. A detailed explanation is presented in File S2. Constraining reactions based on gene expression levels is a common practice in genome-scale metabolic modeling. In this case, reactions have been constrained only if all the genes within the GPR have an expression value above 10. This means that the expression level of all genes associated with a given reaction must be greater than 10 in order to constrain the reaction. The threshold of 10 is chosen as it is assumed to be above the noise level of gene expression data, which can be influenced by technical and biological variability.

“map_transcriptome_data()” requires four positional arguments: 'model', 'transcriptomeData', 'threshold_abundance', and 'max_bound', with 'model' being the genome-scale model; 'transcriptomeData', a dictionary containing the gene IDs (as written in the genome-scale model), as keys and the transcription levels as values; 'threshold_abundance', the threshold to consider that a gene is expressed rather than be noise (for example, 10 as recommended in DESeq2 documentation); and 'max_bound', the value of the upper bounds when there is no restriction (for example, in the Yeast8 model, the lower and upper bounds range from −1,000 to 1,000 so the 'max_bound' is 1,000). This function can be installed using python3 pip or downloaded at https://github.com/Sookie-S/Mapping-of-transcriptome-data-to-genome-scale-model-reactions. A description of how to install and use this function is provided in File S2.

### Media and yeast culture

*S. pastorianus* CBS1513 and *S. cerevisiae* BY4743 strains were used in this study. Starter cultures were generated by inoculating a 5-mL volume of YPD (yeast extract, 10 g/L; peptone, 20 g/L; glucose, 20 g/L) with cells and by incubating at 22°C with shaking at 200 rpm overnight. Biomass was recorded via optical density (OD) measurement at 600 nm with a BioSpectrometer (Eppendorf).

### PCR–based generation of gene knockout mutants

For gene deletions in *S. pastorianus*, a PCR-based fragment fusion method approach was used (47, 48). Gene knockout cassettes were created by amplification of and fusion of three fragments, 1-kb homolog region upstream and downstream of the target gene, and the selectable marker. Primers used for amplification are listed in Table S8. Strains were transformed using the LiAc/SS carrier DNA/PEG method (49), and transformants were confirmed by analytical PCR.

### HPLC analysis

*S. pastorianus* was grown in 200 mL in synthetic minimal media (SD medium: 1X Yeast Nitrogen Base [YNB]; 1X Complete Supplement Mixture [CSM], both Formedium; 2% (wt/vol) glucose) after inoculation with a washed overnight culture to reach an initial OD of 0.1. The glycerol production was calculated when the glucose was fully consumed, at day 7 for 30°C and at day 10 for 13°C. The metabolites were measured by high-performance liquid chromatography (HPLC) using a 1260 Infinity II LC System with a Refractive Index Detector (Agilent). A 300  ×  7.8 mm Hi-Plex HPLC Column (Agilent) was equilibrated with 5 mM H_2_SO_4_ in HPLC-grade water at 55°C at a 0.8 mL/min flow rate. Prior to analysis, the samples were filtered (pore size, 0.45 µm). Quantification was achieved using a refractive index detector (RID). Calibration curves from authentic standards were used to quantify the metabolites produced.

### GC-MS analysis

Volatile compounds were detected and analyzed by GC-MS. Cells were grown in 5 mL of SD complete, SD lacking leucine (SD-LEU), and SD with 100 mg/L leucine on 20-mL vials added with 25 µL of the internal standard 2-octanol (2.5 mg/L) after inoculation with a washed overnight culture to reach an initial OD of 0.1. All the cultures grew at 20°C for 10 d.

Samples were analyzed on an Agilent 7890B Gas Chromatograph (GC) paired with an Agilent 5977B Series Mass Selective Detector (MSD) and operated with a Gerstel MPS dual head system. Vials containing the sample were incubated (30°C, 10 min) to preconcentrate volatile analytes into the headspace, followed by extraction for 5 min with a 100-μm polydimethylsiloxane (PDMS) fiber and injection into the front inlet. The GC separation was performed using an Agilent VF-5MS column (30 m × 25 mm × 0.25 μM) with a flow rate of 1 mL·min^−1^. The oven gradient was 40°C held for 2 min, 15°C·min^–1^ to 300°C and held for 5 min. The total run time for each sample was 24.3 min. The inlet was set at 280°C, the transfer line was kept at 300°C, the electron impact (EI) source at 230°C, and the quadrupole at 150°C. The MSD mass range scanned was *m/z* 40–400.

The acquired data were processed using LECO ChromaTOF. Peak picking, peak annotation, and statistic confirmation were performed, and peak identification was completed by checking the linear temperature programmed retention index (LTPRI), which is available in the NIST RI database. Inter-measurements peak alignment was performed based on the retention times and mass spectrum. An inter-class comparison was performed between sample class and blank class to eliminate artifact compounds.

## Data Availability

iSP_1513 genome-scale metabolic model is available on GitHub at https://github.com/Sookie-S/Genome-scale-metabolic-model-of-S.-pastorianus-CBS-1513-iSP_1513-. The python3 library developed to map transcriptome data into a genome-scale metabolic model reactions, to enable the imposition of restrictions that accurately represent the experimental conditions being investigated, is available at https://github.com/Sookie-S/Mapping-of-transcriptome-data-to-genome-scale-model-reactions.

## SUPPLEMENTAL MATERIAL

Supplemental Figures msystems.00429-24-s0001.png Figures S1 and S2.

Supplemental Files msystems.00429-24-s0002.docx Supplemental figure legends and Files S1 and S2.

Table S1 msystems.00429-24-s0003.xlsx Inconsistencies between the gene and protein annotation.

Table S2 msystems.00429-24-s0004.xlsx List of genes removed from Yeast8 model.

Table S3 msystems.00429-24-s0005.xlsx Reactions that could be carried out by other protein isoforms.

Table S4 msystems.00429-24-s0006.xlsx Genes and reactions from *S. pastorianus* Kbase draft model.

Table S5 msystems.00429-24-s0007.xlsx Essential genes and reactions in SD + 2% glucose medium predicted by iSP_1513 and Yeast8 models.

Table S6 msystems.00429-24-s0008.xlsx Expression of genes involved in the pentose phosphate pathway.

Table S7 msystems.00429-24-s0009.xlsx GSMM reactions constrained according to the medium used.

Table S8 msystems.00429-24-s0010.xlsx Primers used for the PCR-mediated gene knockouts.

ASM does not own the copyrights to Supplemental Material that may be linked to, or accessed through, an article. The authors have granted ASM a non-exclusive, world-wide license to publish the Supplemental Material files. Please contact the corresponding author directly for reuse.
