# Supplementary figures and images for: Development of a genome-scale metabolic model for the lager hybrid yeast S. pastorianus to understand the evolution of metabolic pathways in industrial settings
Source: mSystems. 2024 May 31;9(6):e00429-24. doi: 10.1128/msystems.00429-24 (PMC11237392; doi:10.1128/msystems.00429-24)

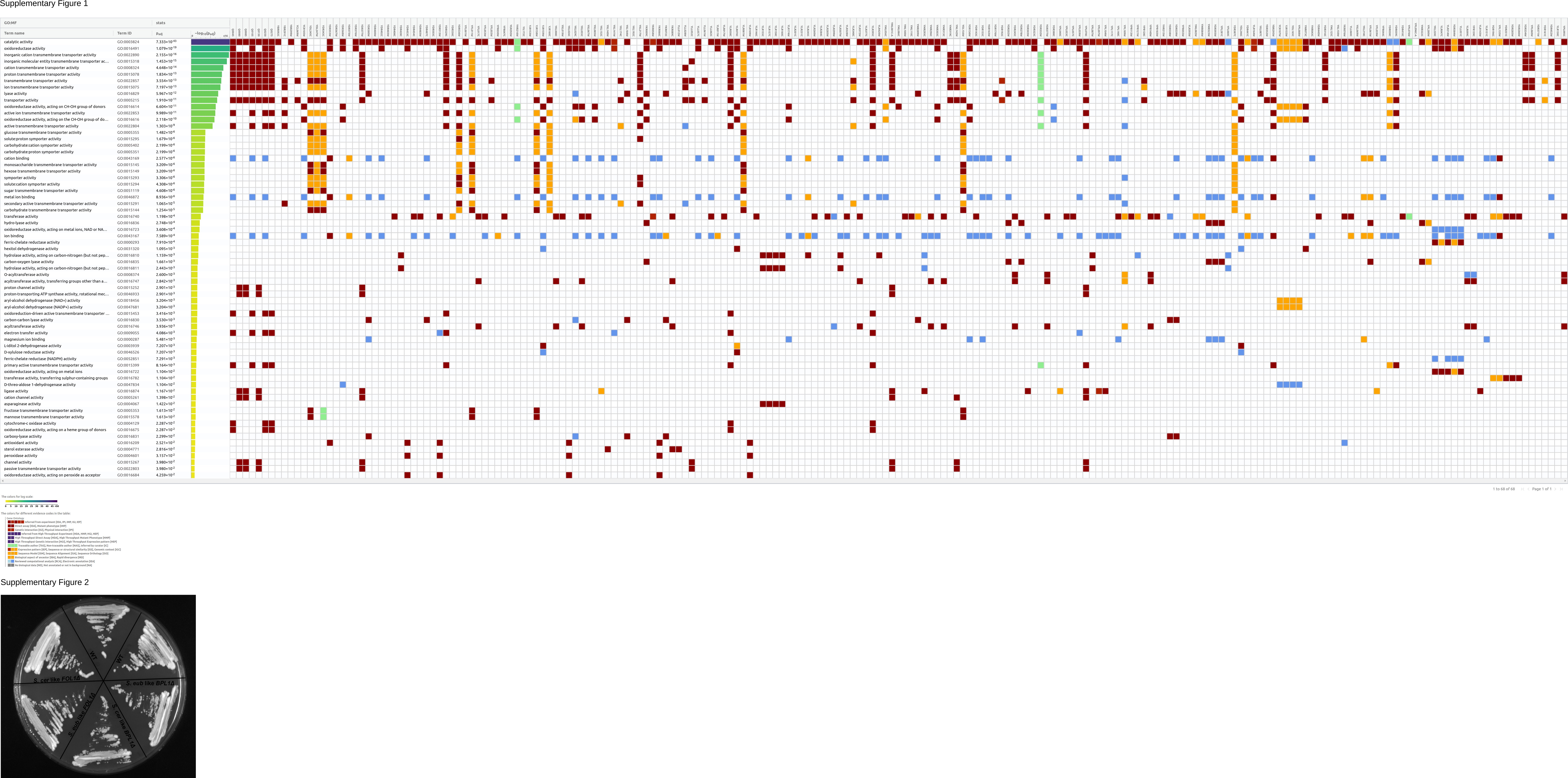

Supplement: Supplemental Figures — Figures S1 and S2. [file msystems.00429-24-s0001.png]
