# Supplementary material for: Development of a genome-scale metabolic model for the lager hybrid yeast S. pastorianus to understand the evolution of metabolic pathways in industrial settings
Source: mSystems. 2024 May 31;9(6):e00429-24. doi: 10.1128/msystems.00429-24 (PMC11237392; doi:10.1128/msystems.00429-24)
Supplement: Supplemental Files — Supplemental figure legends and Files S1 and S2. [file msystems.00429-24-s0002.docx]

**SUPPORTING FIGURES LEGEND**

**Supplementary Figure 1:** GO term enrichment (molecular function) of the 56 *S. cerevisiae* specific genes that were removed from Yeast8 model.

**Supplementary Figure 2: Growth in YPD agar of *S. pastorianus* CBS 1513 wild type (WT), and *S. pastorianus* heterozygote mutant strains carrying the deletion of *S. cerevisiae*-like *FOL1* (“S. cer like *FOL1*△”), *S. eubayanus*-like *FOL1* (“S. eub like *FOL1*△”), *S. cerevisiae*-like *BPL1* (“S. cer like *BPL1*△”) and *S. eubayanus*-like *BPL1* (“S. eub like *BPL1*△”).**

**Supplementary File 1**

**Genes, reactions and metabolites added to the iSP_1513 model**

The cytosolic reaction r_4712 AMP nucleosidase reaction, supported by a protein complex composed by three subunits, has been added to iSP_1513.

H_2_O (c) + AMP (c) --> Adenine (c) + alpha-D-Ribose 5-phosphate (c) (r_4712)

All the genes supporting this reaction are already present in the model except for the *S. eubayanus*-like gene SPGP0R01370, which has been added.

Three nitrilase reactions, r_4713, r_4714 and r_4715, supported by a protein complex composed by three subunits (*NIT1*, *NIT2* and *NIT3*) were added to iSP_1513.

2.0 H_2_O (c) + Indole-3-acetonitrile (c) --> Indoleacetate (c) + NH_4_^+^ (c) (r_4713)

2.0 H_2_O (c) + 2-Aminopropanenitrile (c) --> L-Alanine (c) + NH_4_^+^ (c) (r_4714)

2.0 H_2_O (c) + 4-Amino-4-cyanobutanoic acid (c) --> L-Glutamate (c) + NH_4_^+^ (c) (r_4715)

The metabolites Indole-3-acetonitrile, 2-Aminopropanenitrile and 4-Amino-4-cyanobutanoic were added to the model as they are metabolites of the reactions r_4713, r_4714 and r_4715, respectively. The *S. cerevisiae*-like gene SPGP0DD00150 (NIT1_Scer) was also added to the model, while *NIT2* and *NIT3* are already present in the model.

The reaction acetate reversible transport via proton symport r_4716 has been added alongside the *S. cerevisiae*-like gene SPGP0AE00950 (*BPH1*_Scer) that supports it.

Acetate (e) + H^+^ (e) <=> Acetate (c) + H^+^ (c) (r_4716)

The GPR of the dolichyl-phosphate-mannose--protein mannosyltransferase reaction r_0362 was updated. The *S. cerevisiae*-like gene SPGP0F00900 (*PMT6*) was added to it forms a dimer with SPGP0G00180 (*PMT4*).

The GPR of the glutamine-fructose-6-phosphate transaminase reaction r_0477 was updated. The *S. cerevisiae*-like gene SPGP0DB02080 and the *S. eubayanus*-like gene SPGP0S02100 were added to the GPR of the reaction.

The GPR of the 1,3-beta-glucan synthase reaction r_0005 was updated was updated with the *S. cerevisiae*-like genes SPGP0DB00800 (*GAS3*_Scer), SPGP0K00380 (GAS4_Scer), SPGP0K01360 (GAS5_Scer) and the *S. eubayanus*-like genes SPGP0T00270 (GAS1_Seub), SPGP0N00740 (GAS2_Seub), SPGP0P02990 (GAS4_Seub) and SPGP0P02040 (GAS5_Seub).

The GPR of the asparagine synthase (glutamine-hydrolysing) reaction r_0211 was updated with the *S. eubayanus*-like gene SPGP0DX00470_Seub.

The GPR of the endopolygalacturonase reaction r_ 0365 was updated with the *S. eubayanus*-like gene SPGP0R03420_Seub.

The GPR of the D-mannose transport, D-fructose transport, D-glucose transport and D-galactose transport reactions r_ 1139, r_1134, r_1166, r_1135 respectively were updated with the *S. pastorianus*-specific genes SPGP0B01550_Spas and SPGP0R03480_Spas.

The GPR of the maltose transport reaction r_1227 was updated with the *S. eubayanus-*like and  *S. pastorianus*-like genes SPGP0DN00100_Seub and SPGP0P03210_Spas.

The GPR of the homocysteine S-methyltransferase reaction r_0544 was updated with the *S. pastorianus*-like gene SPGP0U02250_Spas.

The GPR of the 6-phosphogluconolactonase reaction r_0091 was updated with the *S.cerevisiae*-like genes SPGP0M03420 (SOL1_Scer) and SPGP0AE01280 (SOL2_Scer), and *S. eubayanus*-like gene SPGP0DV00410 (SOL1_Seub).

The GPR of the phosphoglycerate mutase reaction r_0893 was updated with the *S. cerevisiae*-like genes SPGP0C02080 (GMP2_Scer) and SPGP0K01120 (GMP3_Scer).

The GPR of the V-ATPase Golgi and V-ATPase vacuole reaction r_1085 and r_1086 respectively were updated with the *S. pastorianus*-specific gene SPGP0P01340 (VMA10_Spas)

The GPR of the chitin deacetylase reaction r_0271 was updated with the *S. eubayanus*-like gene SPGP0N01030.

**Supplementary File 2**

**Description of the algorithm to map transcriptome data.**

The function "map_transcriptome_data" classifies the associations described in the Gene-Protein-Reaction (GPR) rules based on their Boolean relationships, namely OR, AND, and a combination of both. In cases where a reaction is supported by a GPR associated with only one gene, the expression data of that gene is utilized to impose constraints. GPRs that exclusively involve the OR Boolean relationship are constrained by the summation of expression data from the associated genes. Conversely, GPRs featuring solely the AND Boolean relationship are constrained by the minimum expression data among the associated genes. The most complex scenario arises when a GPR involves a combination of OR and AND Boolean relationships. To address such cases in a prioritized manner, a recursive function has been implemented within the "map_transcriptome_data" function.

For example, as illustrated in the following hypothetic GPR (1):

*GENE_ASSOCIATION: ((A or B) and C) or (D and E)) and F* (1)

with *A, B, C, D, E* and *F* different genes, that as genes expression values 100, 70, 30, 60, 90 and 50 respectively.

The recursive function searches for the first block that is not nested within the GPR (1), starting from the end of the GPR, and will calculate the final expression value according to the Boolean operator. In the example, it corresponds to the block (*D* and *E*):

*GENE_ASSOCIATION: ((A or B) and C) or (D and E)) and F* (2)

As the Boolean AND relationship is between D and E, the expression value corresponds to the minimum of their expression values:

*(D and E): min(D, E) = min(60,90) = 60* (3)

The block (D and E) in (2) is next replaced by 60 (3). Recursively, the block that is the least nested becomes (*A* or *B*):

*GENE_ASSOCIATION: ((A or B) and C) or 60) and F* (4)

As the Boolean OR relationship is between A and B, the expression value corresponds to the sum of their expression values:

*(A or B): (A + B) = (100 + 70) = 170* (5)

The block (A or B) in (4) is next replaced by 170 (5). Recursively, the block that is the least nested becomes (170 and C):

*GENE_ASSOCIATION: ((170 and C) or 60) and F* (6)

As the Boolean AND relationship is between 170 and C, the expression value corresponds to the minimum of their expression values:

*(170 and C): min(D, E) = min(170, 30) = 30* (7)

The block (170 and C) in (6) is next replaced by 30 (7). Recursively, the block that is the least nested becomes (30 or 60):

*GENE_ASSOCIATION: (30 or 60) and F* (8)

As the Boolean OR relationship is between 30 and 60, the expression value corresponds to the sum of their expression values:

*(30 or 60): (30 + 60) = 90* (9)

The block (30 + 60) in (8) is next replaced by 90 (9). Recursively, the block that is the least nested becomes (90 and F):

*GENE_ASSOCIATION: 90 and F* (10)

As the Boolean AND relationship is between 90 and F, the expression value corresponds to the minimum of their expression values:

*90 and F: min(90, F) = min(90, 50) = 50* (11)

The block (90 and F) in (10) is next replaced by 50 (11).

*GENE_ASSOCIATION: 50* (12)

The recursive function stops when no more blocks are detected (12).

**Installation instructions for the algorithm to map transcriptome data**

The "map_transcriptome_data" function can be installed using python3 pip as following:

‘python3 -m pip install GSMM_transcriptome_data_mapper’

This function can be used with the following command lines on a python3 terminal:

‘from GSMM_transcriptome_data_mapper import transcriptome_mapper’

‘transcriptome_mapper.map_transcriptome_data(model,transcriptomeData,threshold_abundance,max_bound)’

The script can also be downloaded at <https://github.com/Sookie-S/Mapping-of-transcriptome-data-to-genome-scale-scale-model-reactions>.
